# Contributing reviewers 2022

**DOI:** 10.1186/s40942-023-00516-2

**Published:** 2023-12-12

The Editorial team is grateful to the dedicated reviewers who helped them improve the quality of the articles published in 2022 within the journal International Journal of Retina and Vitreous.

Luis Abad

Mehreen Adhi

Fabio Bom Aggio

Kunihiko Akiyama

Noha Ali

David Almeida

William Anderson

Gabriel Andrade

Tiago Arantes

J. Fernando Arevalo

Albert Augustin

Priscillia Ballalai

Alay Banker

Antonio Marcelo Barbante Casella

Gabriel Barbosa

Guillermo Barriga

Figen Batioglu

Vinicius Bergamo

Laurentino Biccas Neto

Lukas Bisorca-Gassendorf

Marcio Bittar Nehemy

Maria Teresa Bonanomi

Subhendu Boral

Francesco Boscia

David Boyer

Rodrigo Brant Fernandes

Oswaldo Brasil

Leandro Cabral Zacharias

Pedro Carricondo

Nelson Chamma

Felipe Conti

Giulia Corradetti

Elaine Costa

Leonardo Cunha

Leonardo Cunha Castro

Francyne Cyrino

Alessandro Daré

Hudson De Carvalho Nakamura

hilton arcoverde de medeiros

Eduardo Cunha De Souza

Lucas Denadai

Mohit Dogra

Rodrigo Donato Costa

Lilianne Duarte

Pedro Duraes Serracarbassa

Geoffrey Emerson

Gurkan Erdogan

Teodoro Evans

Michel Farah

Joao Felix

Arthur Fernandes

Magno Ferreira

Minoru Furuta

Thomas Gardner

Sunir Garg

Maciej Gawęcki

Andre Gomes

Julio González Martín-Moro

Alexandre Grandinetti

John Helal Junior

Gustavo Heringer

Thomas Hwang

Makoto Inoue

David Isaac

Eliane Jorge

Rodrigo Jorge

Airton Kronbauer

Andres Kychenthal

Gregory Lee

Luiz Lima

Virgilio Lima-Gómez

Anat Loewenstein

Jose Lucas de Souza Filho

Cleide Machado

Ana Paula Maganhoto

Fernando Malerbi

Ahmad Mansour

Flavio Medina

Rodrigo Meirelles

Gustavo Melo

Thais Mendes

Carsten Meyer

Zofia Michalewska

Mihai Mititelu

Nilva Simeren Bueno de Moraes Ambrogini

Fabio Morais

Dario Pasquale Mucciolo

Luis Nakayama

Raja Narayanan

Heloisa Nascimento

Mauricio Nascimento

Carlos Neto

Mohamad Reza Niyuosha

Pradeep Kumar Panigrahi

R. Paranhos

Renato Passos

Fernando Penha

Felipe Pereira

Goran Petrovski

Luisa Pierro

Sergio Pimentel

Fernanda Porto

Rony Preti

Amar Pujari

Jose Pulido

Hugo Quiroz-Mercado

Caíque Ribeiro

Annekatrin Rickmann

Ever Rodriguez

Francisco Rodriguez

Alejandro Rodriguez-Garcia

Luiz Roisman

Jaime Roizenblatt

Assaf Rozenberg

Josmar Sabage

Shohista Saidkasimova

Taiji Sakamoto

Tatsuhiko Sato

Sandeep Saxena

Tarun Sharma

Helio Shiroma

Monica Signorelli

Bradley Smith

Mohamed Soliman

Carlos Eduardo Souza

Osias Souza.

Filippo Tatti

Anderson Teixeira

Eduardo Tomazoni

Marcos Vale

Carlos Veloso

Pradeep Venkatesh

Raul Vianna

Sung Watanabe

Joyce Yamamoto

Marcelo Zas

Focke Ziemssen

